# STEC in the natural environment of Uruguay: genomic surveillance and environmental circulation in the framework of One Health

**DOI:** 10.1128/spectrum.03153-25

**Published:** 2025-11-28

**Authors:** Y. Figueroa, C. Stoletniy, V. Michelacci, D. Montero, J. Silvera, G. Martínez de la Escalera, F. Bertoglio, C. Croci, F. Lepillanca, H. Coitiño, P. Zunino, R. M. Vidal, S. Morabito, C. Piccini, A. Umpiérrez

**Affiliations:** 1 Departamento de Microbiología, Instituto de Investigaciones Biológicas Clemente Estable 113067, Montevideo, Uruguay; 2 Centro de Investigación en Ciencias Ambientales, Montevideo, Uruguay; 3 Department of Food Safety, Nutrition and Veterinary Public Health, Istituto Superiore di Sanità 9289, Rome, Italy; 4 Programa de Microbiología y Micología, Instituto de Ciencias Biomédicas, Facultad de Medicina, Universidad de Chile, Santiago, Chile; 5 Laboratorio de Ecología Microbiana Acuática, Departamento de Microbiología, Instituto de Investigaciones Biológicas Clemente Estable, Montevideo, Uruguay; 6 ONG ECOBIO, Montevideo, Uruguay; 7 INFRAVERDE, Montevideo, Uruguay; Universita degli Studi di Bari Aldo Moro, Valenzano, Bari, Italy

**Keywords:** LAA pathogenicity island, wildlife, environmental STEC, STEC LEE negative, non-O157 STEC

## Abstract

Shiga toxin-producing *Escherichia coli* (STEC) are zoonotic pathogens with significant public health implications. The interplay between humans, domestic animals, wildlife, and environmental reservoirs such as water bodies influences their circulation. This study aimed to evaluate the role of the environment in the dissemination of STEC. Fourteen STEC isolates were recovered from stream water and feces of wild animals collected between 2022 and 2024 in a tourist and wild village-like region in Uruguay. All isolates carried the *stx2* gene, and one also harbored *stx1* and *eae* (*E. coli* O157:H7). The remaining isolates were LEE-negative. Most isolates were susceptible to tested antibiotics. Whole genome sequencing (WGS) revealed a wide diversity of serotypes, sequence types, and virulence gene profiles. Pangenome analysis revealed genomic variability among the analyzed isolates, suggesting the presence of adaptive mechanisms and potential ecological plasticity. The Locus of Adhesion and Autoaggregation (LAA), a pathogenicity island described in some LEE-negative strains, was detected complete in three isolates and partially in three others. Core genome SNP-based phylogenetics and cgMLST analysis (chewBBACA) indicated that the local isolates clustered with strains previously reported from food, livestock, and wildlife across the Americas and Europe, suggesting genetic relatedness. These findings underscore the STEC’s environmental diversity and potential public health risk of STEC circulating in a One Health context. The seasonal detection of the isolates, genomic features, and phylogenetic relationships suggests ongoing interactions between human activity, wildlife, and water sources as key factors in STEC ecology.

**IMPORTANCE:**

Understanding the presence of Shiga toxin-producing *Escherichia coli* (STEC) in the environment is essential to assess potential exposure pathways for animals and humans. In this study, we analyzed STEC isolates recovered from wildlife and environmental samples within a single watershed under a One Health framework. Comparative genomic analyses revealed considerable diversity and the presence of shared virulence determinants, including the *Locus of Adhesion and Autoaggregation* (LAA) pathogenicity island. The detection of related lineages across ecological compartments may suggest possible exchange between environmental and animal reservoirs. These findings provide novel evidence of the environmental presence and dissemination of STEC in this watershed and highlight the importance of integrating genomic surveillance across environmental, animal, and human sectors to better understand the ecological dynamics and public health implications of this pathogen.

## INTRODUCTION

Shiga toxin-producing *Escherichia coli* (STEC) is a zoonotic pathogen mainly associated with livestock, especially bovine, serving as its principal reservoir. Although STEC infects multiple animal species, human cases exhibit the most severe clinical manifestations, ranging from self-limited diarrhea to hemolytic uremic syndrome (HUS). Children, immunosuppressed individuals, and the elderly are at higher risk of developing severe disease (1). The pathogen’s virulence largely depends on Shiga toxins (Stx1 and Stx2), with Stx2 being strongly associated with HUS development (2–4). However, several other virulence factors play crucial roles (5) and contribute significantly to the pathogenic potential of specific strains (6). For example, intimin, encoded by the *eae* gene, is the primary adhesion factor in STEC and is located within the locus of enterocyte effacement (LEE) (7). Despite this, LEE-negative strains can cause severe disease through alternative intestinal adhesion mechanisms. In this context, the adhesion and autoaggregation (LAA) locus represents an important pathogenicity island that contributes to STEC strains adhesion and aggregation properties (8).

Although foodborne transmission remains the most recognized infection route, growing evidence underscores the importance of environmental pathways in STEC epidemiology (9, 10). Animal excrement—not only from ruminants but also from wild birds and mammals—along with inadequate human waste management, contributes to the contamination of water bodies, facilitating the dissemination of STEC into surface and groundwater (11–13). STEC can persist for extended periods in aquatic environments, demonstrating its adaptability to diverse conditions. This persistence underscores that outbreaks may also be linked to the consumption of meat, water, and vegetables contaminated with livestock manure or feces from wildlife inhabiting nearby areas. Additionally, exposure through recreational water activities has been documented as a potential source of infection (14). Despite this, waterborne STEC infections are likely underreported due to challenges in detection, including low bacterial loads, inadequate sampling protocols, and the lack of STEC-specific monitoring in water quality assessments (15).

Conventional methods used to detect STEC in environmental samples have inherent limitations that may lead to underreporting. In many cases, the bacterial load in these types of samples falls below the detection threshold of traditional techniques (16). Isolation of STEC from environmental and wildlife samples was performed using standardized filtration and enrichment protocols. Following isolation, whole genome sequencing (WGS) was employed to provide high-resolution characterization of the isolates, enabling the study of virulence traits, lineage dissemination, and zoonotic potential.

In Uruguay, STEC has been detected in cattle, humans, and food. Some of these strains carry potentially zoonotic genetic variants (17, 18). Although reporting of foodborne illnesses (FBIs) is mandatory (19), the actual incidence of HUS is estimated to be high, similar to other countries in the region (20). Several STEC serogroups have been identified in this context, including O111, O26, O121, O103, and the serotype O157:H7, in both human cases and food sources (21–24). Additionally, serogroups O111 and O103 and other non-O157 strains have been reported in cattle (25, 26) and bovine carcasses (27). Despite the evidence, the role of the environment in disseminating this pathogen remains poorly understood.

In this context, the main objective of this study was to describe the presence and genomic diversity of STEC in a small watershed with tourism, agriculture, and livestock, providing insights into potential environmental reservoirs.

To address the potential role of the natural environment in the local epidemiology of STEC, we investigated its presence in stream water and wildlife feces within a watershed with both rural and urban characteristics. We hypothesized that the natural environment could act as a reservoir for STEC, contributing to the dissemination of this zoonotic pathogen. To explore this possibility, we characterized the recovered isolates in terms of antimicrobial phenotype, virulence potential, and phylogenetic relationships using whole genome sequencing (WGS), core genome SNP-based analysis, and core genome multilocus sequence typing (cgMLST).

## MATERIALS AND METHODS

### Water and fecal sampling

Sampling was conducted bimonthly between 2022 and 2024 (28) within the Los Chanchos stream micro-basin, located in Villa Serrana village, Lavalleja, Uruguay. The micro-basin covers approximately 72 km² of extension, with a permanent population of 270 people and around 4,000 moving there annually, during the high seasons (school holidays and peak tourist activity: April, May, July, September). Land use in the area is dominated by cattle grazing, small-scale agriculture, and forested areas (Fig. S1). Seven sampling sites were selected along Los Chanchos stream micro-basin in Villa Serrana village, Lavalleja, Uruguay. The selection included the most representative land uses and land cover in the basin: grassland with the presence of cattle, urbanization, and forest (Fig. S1). At each sampling point, 1,000 mL of water was collected in sterile bottles for further processing. Additionally, three fresh fecal samples from wild animals were collected per sampling point in the stream surroundings, obtaining approximately 10 g of each sample. The origin of the feces was identified by a person trained for that purpose and verified with camera trap placed at the sampling sites (see Fig. S1). Feces were stored in sterile containers and were kept refrigerated (4°C) until arriving at the laboratory.

### Water and fecal sample processing

From each sampling site, three replicate water samples of 100 mL were filtered using 0.45 µm pore size nitrocellulose filters (Millipore) (29). The filters were placed on m-FC agar plates (HiMedia) and incubated for 18 h at 44.5°C. After incubation, those plates where blue colonies indicated the presence of fecal coliforms (FC) were processed sequentially, as follows: *stx1* and *stx2* screening was performed on FC-positive filters by PCR. Fifty blue colonies per *stx*-positive filter were selected, streaked on tryptic soy agar plates (TSA, Britania) and incubated for 18–24 h at 37°C for *E. coli* isolation.

For the fecal samples, 10 g aliquots were inoculated in tryptic soy broth (TSB, Britania) at a 1/10 dilution and incubated at 37°C for 18–24 h. After enrichment in TSB, aliquots were streaked onto MacConkey agar plates (Oxoid), and up to 50 Lactose-positive (pink) colonies from three agar plates were selected and transferred to TSA (18–24 h at 37°C) to obtain isolates.

### Characterization of the isolates

Up to 50 colonies from TSA plates, from each collected sample (water and feces) were selected and grouped into five pools of 10 colonies each. DNA extraction was performed using the heat block method (30). Conventional PCR was conducted to partially amplify the *stx1* and *stx2* genes (31). Positive pools were further analyzed by individual PCR to identify *stx1* and/or *stx2* positive colonies. Confirmed *stx*-positive isolates were identified as *E. coli* based on conventional biochemical tests and molecular confirmation by 16S rRNA gene amplification (32).

Antibiotic resistance was determined using the Kirby-Bauer disk diffusion method according to CLSI guidelines (2025) (33). The antibiotics tested included ampicillin (AMP), ciprofloxacin (CIP), nalidixic acid (NA), trimethoprim-sulfamethoxazole (STX), enrofloxacin (ENR), gentamicin (GN), tetracycline (TE), cefuroxime (CXM), amikacin (AK), fosfomycin (FOT), amoxicillin-clavulanic acid (AMC), cefepime (FEP), ceftriaxone (CRO), cefoxitin (FOX), ceftazidime (CAZ), and imipenem (IMI). For molecular characterization, PCR was performed to detect genes encoding intimin (*eae*), enterohemolysin (*ehxA*), autoagglutinating adhesin (*saa*), and the serogroups most frequently associated with human STEC outbreaks (O157, O26, O103, O145, O111, O121, O45) (25).

### DNA extraction and genome sequencing of STEC isolates

Fourteen strains belonging to all sampling points were selected, and DNA extraction was performed using the Monarch kit, following the manufacturer’s protocol (New England Biolabs). Whole-genome sequencing (WGS) was conducted using the Illumina NovaSeq 6000 platform (Illumina, San Diego, USA) at MicrobesNG. A paired-end sequencing protocol with short reads of 250 bp was employed (34).

### Basic bioinformatics analysis

Using the reads provided by the sequencing company, bioinformatics analyses were conducted on the Galaxy Aries platform following a protocol established by the EU Reference Laboratory for *E. coli* at the Istituto Superiore di Sanità (35, 36). The quality of the reads was assessed using FastQC (37). Trimming was performed using the FastQ Positional and Quality Trimming tool (38), using a minimum Phred quality score for right-side trimming of 25, a left-side trimming of 20, and an average Phred quality score for right-side trimming of 27. Kmerfinder was used to verify the purity of the reads (39). Genome assembly was performed using SPAdes Genome Assembler (40). The obtained assemblies were filtered using the Filter SPAdes Repeats tool, and the assembly quality was evaluated using QUAST (41). The trimmed reads were used to characterize the genomes with *E. coli* Virulotyper, SeroTypeFinder, Shiga toxin typer (42), and ABRicate with ResFinder database (43). To identify the pangenome of the 14 bacterial isolates, a genomic annotation was performed using Prokka v1.13.7 (44).

The presence or absence of virulence-associated loci was assessed based on Prokka annotations and Virulotyper outputs. The absence of the Locus of Enterocyte Effacement (LEE) was inferred from the lack of its main structural and regulatory genes (*eae*, *ler*, *tir*, *esc*, and *esp*), which together define this pathogenicity island (45).

The phylogroup of each *E. coli* genome was determined *in silico* using the extended Clermont scheme (46). Briefly, the presence/absence of the genetic markers *chuA*, *yjaA*, and *TspE4.C2* was assessed by BLASTn (v2.15.0+) against reference sequences. Phylogroups were assigned as follows: A (*chuA*^−^), B1 (*chuA^+^*, *yjaA*^−^, *TspE4.C2*^−^), B2 (*chuA^+^*, *yjaA^+^*, *TspE4.C2*^−^), D (*chuA^+^*, *yjaA^+^*, *TspE4.C2^+^*), and E (*chuA^+^, yjaA*^−^, *TspE4.C2^+^*).

The LAA pathogenicity island was detected using the Geneious platform, following the protocol described by Montero et al. (47, 48). As a reference, a fully closed genome of STEC O91:H21 was used, which contains the complete LAA pathogenicity island in a single contig (strain: P15-385, accession: JAEKGL000000000) (49). The LAA island is in the genomic region between positions 3,867,470 and 3,954,019 bp. The strains under study were arranged relative to the reference strain using the MCM algorithm. Once the sequences were ordered, an alignment was performed using the Mauve Progressive software, allowing the identification of collinear blocks (LCBs) corresponding to the aligned regions (50). After identifying the LAA pathogenicity island, the presence of the modules associated with this island and their genomic contexts was evaluated. The following procedure was followed: the aligned genomes were concatenated, and the fragment containing the pathogenicity island was extracted and saved as a separate file in .gbk format. Finally, the Easyfig tool was used to visualize the similarities between the fragments analyzed (51).

### Phylogeny of LEE-Negative STEC strains

In addition to the 14 genomes analyzed in this study, 92 LEE-negative STEC genomes were retrieved from the NCBI database. These genomes were selected based on the following criteria: (i) regional STEC genomes and (ii) STEC genomes isolated from environmental matrices or wildlife, comparable to those included in our study (Table S1). To determine the phylogenetic relationships among the strains, a core gene alignment was performed using the Roary pangenome pipeline (52), categorized genes into core (conserved in all isolates), accessory (variably present), and unique genes, enabling insights into evolutionary dynamics, virulence, and ecological adaptation. Only sites with variations (SNPs) were extracted from the core genome alignment using the “Finds SNP sites from a multi-FASTA alignment file” tool (53). Subsequently, a SNP-based phylogenetic tree was generated using IQ-TREE Phylogenomics (54). Tools such as FigTree (55) and iTOL were used to visualize the tree (56).

To complement the SNP-based phylogenetic analysis, a cgMLST approach was applied using chewBBACA v2.8.5 (57). Allele calling was performed with the *Escherichia/Shigella* schema (INNUENDO project), which includes 2,360 loci. The allele distance matrix was generated with the Allele Distance module, and pairwise comparisons between local and reference genomes were used to assess relatedness. Isolates differing by <10 alleles were considered to be closely related (58).

## RESULTS

### Phenotypic characterization: typing the STEC collection

A collection of 14 STEC isolates was obtained from environmental samples collected in Villa Serrana, Uruguay, including stream water and feces from wild animals (Table 1). Most isolates were detected during periods of increased tourist activity, such as Easter Week (April–May), winter holidays (July), and the school recess (September).

**TABLE 1:** Origin, site of collection, phenotypic and genomic characterization of STEC isolates from wildlife feces and water samples^*a*^

Strain	Date	Land use and cover	Tourist activity	Host/origin	Phenotypic resistance profile	*stx* subtype	Serotype	Phylogroup	Sequence type
F112-CAVS1	Sep-22	Grassland /cattle	High	*Mazama gouazoubira^b^*	DS (AK)	*stx2d*	O15:H27	A	2388
F114-CAVS1					–^*e*^	*stx2d*	O15:H27	A	2388
F113-CBVS9		Urbanization		*Sus scrofa^c^*	–	*stx1a* *, stx2c*	O157:H7	B1	11
11F1-CBVS9	Nov-22	Urbanization	Low	*Sus scrofa*	DS (AK)	*stx1a* *, stx2a*	O-:H21	A	1248
11F2-CBVS9					–	*stx1a* *, stx2a*	O-:H21	A	1248
15F2-CBVS10	May-23	Urbanization	High	*Sus scrofa*	–	*stx2a*	O163:H19	A	679
15F4-CBVS10					DS (AK)	*stx2a*	O163:H19	A	679
BI5	Jul-22	Urbanization	High	Los Chanchos stream	–	*stx2d*	O174:H21	E	677
CAVS34	Sep-22	Grassland /cattle	High		–	*stx2a*	O120:H1	D	104
CAVS59		Urbanization			–	*stx2c*	O6:H34	A	7616
CBVS84		Forest			–	*stx2a*	O15:H49	A	N/A^*d*^
CBVS85					–	*stx2a*	O116:H49	A	2520
CAVS18	Nov-22	Grassland /cattle	Low		–	*stx2b*	O-:H16	A	295
CAVS123	Aug-24	Grassland /cattle	Low		–	*stx2c*	O91:H7	A	677

^*a*^ Decreased sensitivity (DS), Amikacin (AK).

^*b*^ *Mazama gouazoubira* (deer).

^*c*^ *Sus scrofa* (wild boar).

^*d*^ N/A indicates allele missing.

^*e*^ “–” indicates none resistance detected.

All the isolates were confirmed as *E. coli* and tested positive for the *stx2* gene. The *stx1* and *eae* genes were detected in only one isolate (F113). None of the remaining isolates carried other LEE-associated genes (*ler, tir, esc, esp*), confirming the absence of this pathogenicity island (45). Isolates were sensitive to all tested antibiotics. However, reduced susceptibility to AK was detected according to the criteria established by the CLSI 2025 (Table 1).

### Genomic characterization of environmental STEC

#### Pangenome analysis

A pangenome analysis was performed to explore the genetic diversity and possible adaptive mechanisms of STEC environmental isolates. The average genome length was 4,872,997 bp, with an average GC content of 51%, typical of the *E. coli* species. A total of 9,601 genes were identified, including 1,830 core genes (shared by 99%–100% of isolates), zero soft-core genes (95%–99%), 3,364 shell genes (15%–95%), and 4,407 cloud genes (≤15%).

#### Genome analysis

As determined by PCR, all isolates carried the Shiga toxin 2 (*stx2*) gene, while one isolate (F113) also encoded the Shiga toxin 1 (*stx1*). The subtypes *stx1a, stx2a, stx2b, stx2c*, and *stx2d* were identified. The *eae* gene was detected only in F113 isolate, corresponding to the O157:H7 serotype. Four serotypes and four sequence types were identified in the isolates obtained from fecal samples, while seven serotypes and five sequence types were detected in the water isolates. All 14 STEC genomes were assigned to *E. coli* phylogroups using the extended Clermont scheme (Table 1). The majority belonged to phylogroup A (*n* = 11), while single isolates were classified into phylogroups B1 (*n* = 1), D (*n* = 1), and E (*n* = 1).

**Fig 1:**
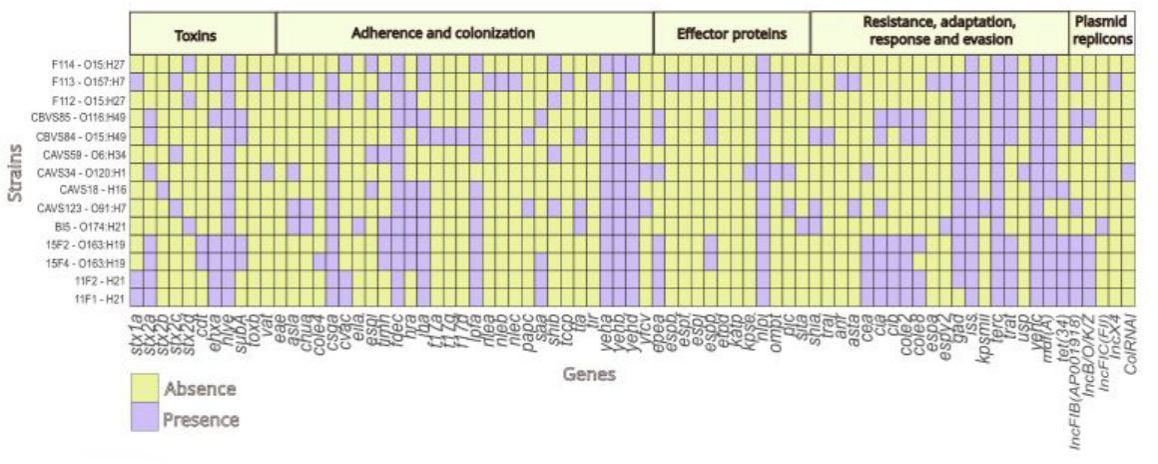
Heatmap of presence/absence of virulence, toxins, adherence, adaptation, antimicrobial resistance, and protein effectors genes in the analyzed genomes. Each row represents a genome, and each column corresponds to a specific gene. The presence is indicated in purple, while the absence is shown in green. Figure made with RStudio.

Diversity of virulence genes was identified, including those related to toxin production, adhesion, colonization, and others associated with antimicrobial resistance, adaptation, and in the response to and evasion of adverse environmental conditions, as detailed in Fig. 1.

#### Antimicrobial resistance genes and pathogenicity determinants

*In silico* analysis identified *mdfA* in 13 out of 14 isolates, associated with macrolide resistance. The *tet(34)* gene was also detected in five isolates. The most frequently identified plasmid replicon was IncFIB(AP001918) (nine isolates), followed by IncB/O/K/Z (five isolates). Other replicons included IncFIC(FII), IncX4, and ColRNAI, each found in a single isolate (Fig. 1).

**Fig 2:**
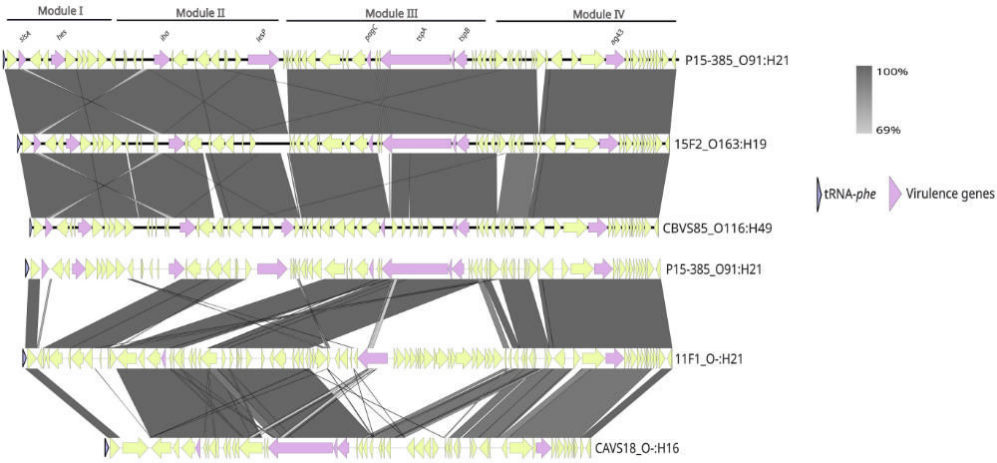
Comparison of the genetic structure of LAA pathogenicity island present in STEC from water and feces. 15F2 and CBVS85 are shown with a complete LAA island (four modules). Isolates with partial LAA island (two modules for CAVS18 and three modules for 11F1) are also shown. Arrows represent predicted genes and direction of transcription. The names of the major virulence genes present in the island are shown (purple). Conserved regions between islands are shaded gray, and color intensity indicates nucleotide identity levels (69%–100%). Figure made with Easyfig.

Genome alignment with closed genome of P15-385 strain (O91:H21) revealed the presence of the LAA pathogenicity island in STEC isolates from feces and water. Out of 14 genomes, 3 harbored the complete LAA island, while 3 additional genomes contained a partial LAA island. The remaining eight genomes showed no evidence of LAA, due to various reasons detailed below. The LAA island was identified downstream of the *pheV* gene. As shown in Fig. 2, isolates 15F2, 15F4, and CBVS85 harbored the complete pathogenicity island, with all four conserved modules and the presence of the main proteins described by Montero et al. (48, 59). In contrast, 11F1, 11F2, and CAVS18 contained an incomplete LAA island. Specifically, the 11F1 and 11F2 genomes harbored modules II, III, and IV, while the CAVS18 genome harbored only modules III and IV. On the other hand, in F112 and F114 genomes, an insertion different from the LAA island was identified (data not shown). The CAVS34 and CAVS59 genomes showed no evidence of the LAA island. As expected, the F113 genome corresponds to LEE-positive STEC and does not contain LAA. Finally, BI5, CAVS123, and CBVS84 genomes have multiple contigs in the island region, which prevented a precise analysis; therefore, these isolates were not considered in the LAA island evaluation.

#### Phylogenetic relationship of LEE-negative isolates

**Fig 3:**
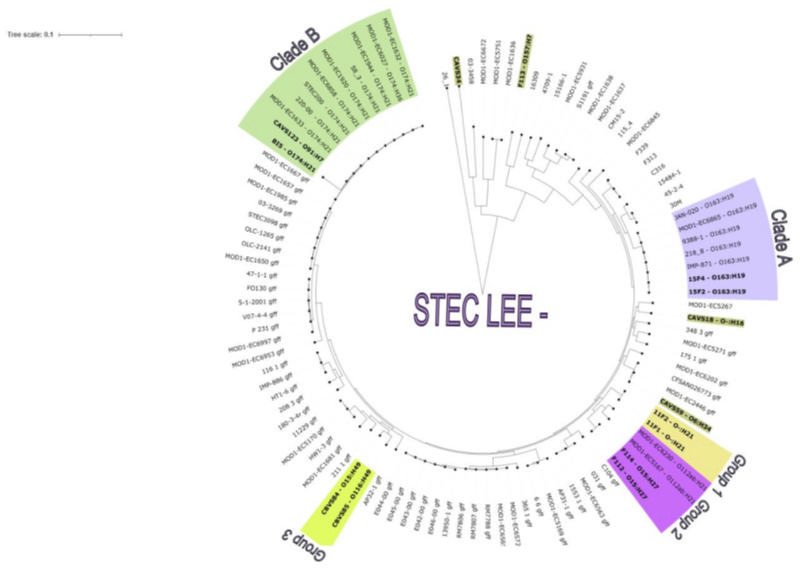
Phylogenetic relationships among LEE-negative STEC isolates. In addition to the 14 isolates recovered in this study (including strain F113, corresponding to O157:H7), 92 previously published LEE-negative STEC genomes were used to construct a maximum likelihood phylogenetic tree (midpoint-rooted) based on genome-wide SNPs. Colors indicate different clades or groups. Isolates from this study are shown in bold. Tree generated using FigTree/iTOL.

As observed in the phylogenetic tree (Fig. 3), 15F2 and 15F4 *Sus scrofa* (wild boar) isolates (O163:H19) obtained in 2023, clustered within Clade A together with other O163:H19 isolates from diverse origins, including meat products in the USA (2006), Chile (undated), and Uruguay (2004), and from bovine feces in the USA (1996, 1999) (48).

Isolates BI5 (O174:H21) and CAVS123 (O91:H7), recovered from the stream in 2022 and 2024, respectively, formed Clade B along with isolates from diverse sources and time periods: human feces in Canada (1988), food and bovine feces in the USA (1989–2010), bovine feces in Chile (undated), human feces in the Netherlands (2013), and a HUS case in Germany (2000) (48) (Fig. 3). Except for CAVS123, all isolates belonged to serogroup O174 and harbored the LAA island; however, CAVS123 shared the H21 flagellar antigen with them. Although genome fragmentation prevented confirmation of the LAA island in CAVS123.

Group 1 included isolates 11F1 and 11F2 (O–:H21), genetically identical and recovered from *Sus scrofa* (wild boar) in 2022. These isolates shared a common ancestor with Group 2—formed by F112 and F114 (O15:H27), two almost identical isolates from *Mazama gouazoubira* (deer) feces (Fig. 3). Group 2 isolates (F112 and F114) showed close relatedness to those from the small intestine of *Odocoileus virginianus* (deer) (indicated in violet in Fig. 3). Notably, Group 1 strains carried three modules of the LAA island, while Group 2 strains lacked it (48) (Fig. 3).

Additionally, CBVS84 and CBVS85 isolates formed Group 3, with a recent common ancestor. Both shared the H49 antigen and were isolated in the same year and month. In contrast, CAVS18 (November 2022) and CAVS123 (August 2024), collected from nearby locations, showed genetic divergence despite geographic proximity (Fig. 3).

Finally, isolate F113 (O157:H7) exhibited a high genetic distance from most of the analyzed isolates, showing marked genetic distance from most other isolates (Fig. 3). It was the only LEE-positive isolate included in the phylogenetic analysis, used as a comparative reference for a high-pathogenicity STEC lineage. However, it did not cluster as an external clade in relation to the LEE-negative genomes considered in this analysis.

Furthermore, cgMLST analyses showed close allelic similarities between local strains and previously published STEC genomes from diverse countries and sources (48). For example, isolate 15F2 differed by 14–28 alleles from strains in clade A (48), while isolate CAVS123 showed 12–32 allele differences with strains in clade B (48).

## DISCUSSION

In this study, we demonstrated the presence and genomic diversity of STEC in environmental and wildlife samples from a rural-touristic region. Our findings indicate the presence of STEC in both water and wildlife feces, with diverse virulence and antimicrobial resistance genes and pathogenicity islands, including LAA island. STEC detection and isolation were more frequently observed during months corresponding to school holidays and peak tourist activity in Villa Serrana (April, May, July, September). This pattern raises the hypothesis that ecological and anthropogenic factors may influence the environmental dynamics of STEC although additional data are needed to confirm such associations. These findings underscore the potential role of natural environments as reservoirs and transmission interfaces for STEC and reinforce the value of integrated studies. Seasonality, wildlife–human interactions, and land use are possible modulators of STEC occurrence and dissemination in shared environments.

### Virulence and environmental adaptation genes

*E. coli* have acquired genotypic and phenotypic traits enabling adaptation to diverse environments (57, 58).

All 14 isolates carried the *stx2* gene, and one also was *stx1^+^*. Subtypes *stx1a*, *stx2a, stx2b, stx2c, stx2d,* and *stx1a* were detected, all associated with severe disease (59, 60). Subtypes *stx1a, stx2a, stx2c*, and *stx2d* also are the most prevalent in STEC from cattle (61). Among free-ranging ruminants, Cervids (including *Mazama gouazoubira*) are important reservoirs, with *stx2b* (detected here) being predominant and linked to human infection (62). According to the reviewed literature, multiple Stx2 subtypes have been detected in deer to date. This suggests that these isolates could contribute to water contamination and, under certain circumstances, represent a potential outbreak risk to human health (63).

STEC was also found in *Sus scrofa* (wild boar) (3, 64). It is worth noting that in Uruguay, *Sus scrofa* hunting is promoted by the Ministry of Environment due to its status as an invasive species (65). However, meat, intended for human consumption, is not included in the National Food Safety Regulations (66), which could pose as an additional transmission route of STEC to the population.

Hemolysin genes *hlyE* and *ehxA* were detected in all isolates and in five *Sus scrofa* isolates, respectively, promoting erythrocyte lysis and bacterial proliferation (67–69). Additional toxins included *cdt*, *subA* (with one isolate also carrying *saa*), and *toxB*, consistent with previous reports for LEE-negative STEC (70–72). Adhesion and colonization factors identified were *fdeC*, *yehB*, *yehA*, *yehD*, *lpfA*, *csgA*, and *iha*, while the *eae* gene was detected only in the single LEE-positive strain (F113). Genes *lpfA*, *iha,* and *fdeC* enhance adhesion and invasion, particularly when co-expressed with other fimbrial components, while *csgA* and *nlpI* support biofilm formation and resistance to environmental stress (73–81).

Finally, multiple genes involved in environmental persistence and host immune evasion were detected, including *iss* (bloodstream survival), *terC* (tellurium adaptation), and *gad* (acid response) (82–84). Together, these factors may contribute to the capacity of LEE-negative STEC to survive and persist across diverse ecological niches. However, the presence of these genes does not guarantee its expression; further studies should be performed to confirm these assumptions.

### Phenotypic and genomic antibiotic resistance

With respect to antibiotic resistance, the *mdf(A*) gene was detected in 13/14 isolates. It codifies to a common efflux pump in *E. coli*, whose expression is associated with resistance to macrolides, as well as lincosamides and streptogramin B (MLS) (85). Additionally, the *tet(34)* gene was detected in five isolates. Since its discovery until today, tetracyclines have been used intensively in agriculture as well as in human and veterinary clinical settings (86). Despite studies questioning its classification, the *tet(34)* gene has been classified as a determinant of tetracycline resistance by enzymatic inactivation (87, 88). In addition, this determinant is not mentioned in a recent study (89). Despite this uncertainty, the database used in this study continues to include *tet(34)* in its latest update (90). In our analysis, the finding of the *tet(34)* resistance gene was not associated with phenotypic resistance.

### Report on the LAA pathogenicity island

According to the literature consulted, this is the first report describing the LAA island in Uruguay. Although a previous study inferred its presence based on the partial detection of some virulence genes involved (27), the presence of the LAA island in STEC was not confirmed. Due to its modular nature, the LAA island can be found in different isolates as a complete structure (with all four modules) or incomplete (with one, two, or three modules). Its association with disease has only been documented when the island is complete (48).

We detected the presence of the *hes* gene (module I marker) exclusively in genomes harboring the complete LAA island. This gene encodes a heat-resistant agglutinin (Hra family) involved in colonization processes such as cell adhesion and aggregation (48). The complete LAA island was identified in the O163:H19 (15F2 and 15F4) and O116:H49 (CBVS85) serogroups, both previously described in regional isolates from Uruguay, Argentina, and Chile, primarily from cattle (48). Interestingly, the O163:H19 serogroup was also reported in *Sus scrofa* feces from the USA (48). This serogroup was also reported in 2001, lacking the LAA island (48).

In contrast, incomplete LAA islands—as observed in serotype O-:H21 (11F1 and 11F2), which only contain modules II, III, and IV—did not harbor the *hes* gene. This serotype has also been reported in cattle carcasses from Uruguay (27).

From an ecological standpoint, the LAA island encodes factors associated with adhesion and self-aggregation, which may promote biofilm formation and survival in harsh or host-free environments. These properties could confer a selective advantage in ecosystems, particularly aquatic habitats, by enhancing survival by forming protective cellular aggregates. However, it is important to note that the presence of LAA does not necessarily predict pathogenic potential since LEE-negative STEC carrying this island are frequently detected in food and environmental samples but remain poorly studied associated with human infections (91).

### Pan-genome study

The STEC pangenome reflects remarkable genetic plasticity, which is characteristic of its highly dynamic and promiscuous accessory genome. In this study, the mean genome size of the isolates was similar to that reported in environmental *E. coli* from lakes, sewage, and marginal urban areas. This suggests that, although STEC strains possess genetic elements associated with virulence, their overall genome size does not differ significantly from other environmental populations of the species (57).

Within the accessory genome, shell genes include virulence factors, antibiotic resistance genes, and elements related to adaptation to different environments or hosts. Cloud genes represent the most variable component of the pangenome, such as plasmids, phages, and genomic islands. These genes may play a key role in the diversity and evolution of STEC, contributing to new metabolic or virulence capabilities (92). Consistently, the high proportion of variable genes detected in our data set suggests a high genetic diversity, which could reflect adaptations to different environments, hosts, and selective pressures. This diversity likely facilitates the maintenance of STEC in non-human reservoirs and the emergence of novel lineages with distinct genetic features.

Given that the LEE-negative genotype is common among environmental STEC isolates, we focused on strains lacking the LEE pathogenicity island to better understand the epidemiological and evolutionary relevance of these strains within the regional STEC population. We extended our analysis to include regional LEE-negative STEC genomes from environmental and wildlife sources available in public databases.

The phylogenetic analysis of LEE-negative STEC revealed high genetic diversity and allowed the identification of clustering patterns among strains obtained from different hosts and geographical locations. The close relationship between F112 and F114 (*Mazama gouazoubira*) genomes and the labeled isolates (purple color in Fig. 3) obtained from the small intestine of *Odocoileus virginianus* could reveal a phylogenetic link within the *Cervidae* family. This finding reinforces the hypothesis that certain STEC clones could be adapted to specific hosts and share similar ecological niches (93).

Isolates 15F2 and 15F4 (O163:H19) showed a strong relationship with historical isolates of the same serotype from various sources in the Americas. The coincidence with the associated strains (depicted in violet in Fig. 3) suggests a wide geographic spread of this variant. The close relationship with IMP-871, isolated in Uruguay in 2004 from a meat product, could indicate either the maintenance of this serotype in the country or a reintroduction through various transmission routes (48).

On the other hand, the BI5 and CAVS123 isolates, obtained in 2022 and 2024, respectively, clustered with multiple O174:H21 isolates, including human feces (Canada, Netherlands, Germany), bovine feces (Canada, Chile, USA), and food (USA). Notably, the O174:H21 strain BI5 was assigned to phylogroup E—the lineage typically associated with enterohemorrhagic *E. coli* O157:H7. The detection of phylogenetically related strains in diverse hosts and environments supports the idea that STEC can disperse and adapt to different epidemiological chains. The relationship of these isolates with those detected in clinical cases confirms the zoonotic potential of these variants, emphasizing the need for further studies to evaluate their pathogenicity and transmission mechanisms (94).

As for the relationship with the complete or partial presence of LAA island, phylogenetic analysis revealed distinctive patterns among the isolates. On the one hand, isolates 15F2 and 15F4 (O163:H19) showed a close relationship with previously described strains from different geographical origins and sources, consistently harboring the complete LAA island. This suggests that the LAA island has been conserved within the lineage, potentially providing a specific adaptive advantage. In contrast, F112 and F114 (O15:H27) genomes, closely related to *Odocoileus virginianus* isolates, lacked the LAA island. Its absence could indicate an evolutionary loss or a failure in its acquisition. In isolates BI5, CBVS84, and CAVS123 (O174:H21, O15:H49, and O91:H7), the LAA investigation was inconclusive due to genome fragmentation in the insertion region. However, given its phylogenetic relationship to strains on which the island has been identified, it is presumed to be present in its complete form. All together, these results suggest that the presence of LAA may be associated with specific phylogenetic lineages and influenced by ecological factors or selective pressures in different hosts and environments.

A notable finding was the clustering of CBVS84 and CBVS85 isolates, obtained from the same stream area. Despite sharing a recent common ancestor, these isolates exhibit differences in their virulence and adaptation genes, which could indicate diversification driven by environmental pressures or colonization in different ecological niches. In contrast, CAVS18 and CAVS123 isolates showed significant genetic divergence despite their geographical proximity. This suggests that, although both isolates may have shared a common environment, they could have evolved independently or been introduced at different times from different sources. This finding highlights the complex dynamics of STEC isolates and suggests that multiple lineages can coexist and differentiate within the same geographic region in short periods.

Although cgMLST distances exceeded the strict clonal threshold of 10 alleles, the observed differences (12–32 alleles) were comparatively low considering the 20- to 30-year temporal gap. This may indicate limited variability whithin certain STEC lineages, as has been described for O24:H11 ST29 (72). These results suggest that low allelic differences over extended periods may reflect genome stability or the persistence of specific clones across regions. Beyond our data set, integrating cgMLST with SNP-based phylogenetics represents a complementary approach that strengthens resolution and provides a broader framework to understand the evolutionary dynamics of STEC lineages.

The diversity and prevalence of STEC in natural environments and wildlife underscore the need for regular surveillance programs under a One Health approach. Integrating environmental, livestock, and wildlife monitoring could enable early detection of emerging variants and strengthen prevention strategies. Given the discordance between clinical epidemiological data and the frequent detection of STEC in food and environmental reservoirs (91), systematic monitoring becomes crucial to better contextualize the public health significance of these findings. These observatios are in accordance with our actual work; however, the relatively small number of isolate requires future studies with a broader temporal sampling and a higher number of sequences.

### Conclusion

Our findings reveal a diverse population of environmental STEC strains carrying numerous genes that may contribute to adaptive and evolutionary advantages. These strains differ genetically from the classical LEE-positive STEC previously reported in Uruguay, which have been mainly associated with human clinical cases, bovine disease, and foodborne contamination. Although one LEE-positive O157:H7 strain was detected in a *Sus scrofa* specimen, most isolates belonged to alternative lineages, several of which carried the LAA pathogenicity island. The detection of similar genomic features among environmental isolates and those from wildlife and livestock highlights their circulation across different host and ecosystems. These results point to the prevalence of distinct LEE-negative lineages in natural settings and reinforce the importance of continued surveillance within a One Health framework.

## Data Availability

Assembled sequences obtained in this study were deposited in the NCBI database under BioProject PRJNA1219087.

## SUPPLEMENTAL MATERIAL

Figure S1 and Table S1 spectrum.03153-25-s0001.pdf Figure S1: Geographical localization of Villa Serrana village and stream "Los Chanchos" in Uruguay, coverage and land uses, sample sites. Table S1: LEE-negative STEC used to generate the Core genome SNPs tree.

ASM does not own the copyrights to Supplemental Material that may be linked to, or accessed through, an article. The authors have granted ASM a non-exclusive, world-wide license to publish the Supplemental Material files. Please contact the corresponding author directly for reuse.

## References

[1] Koutsoumanis K, Allende A, Alvarez‐Ordóñez A, Bover‐Cid S, Chemaly M, Davies R, De Cesare A, Herman L, Hilbert F, Lindqvist R, Nauta M, Peixe L, Ru G, Simmons M, Skandamis P, Suffredini E, Jenkins C, Monteiro Pires S, Morabito S, Niskanen T, Scheutz F, da Silva Felício MT, Messens W, Bolton D. 2020. Pathogenicity assessment of Shiga toxin‐producing Escherichia coli (STEC) and the public health risk posed by contamination of food with STEC. EFS2 18:e05967. doi:10.2903/j.efsa.2020.5967

[2] Mora A, López C, Dhabi G, López-Beceiro AM, Fidalgo LE, Díaz EA, Martínez-Carrasco C, Mamani R, Herrera A, Blanco JE, Blanco M, Blanco J. 2012. Seropathotypes, phylogroups, Stx subtypes, and intimin types of wildlife-carried, shiga toxin-producing Escherichia coli strains with the same characteristics as human-pathogenic isolates. Appl Environ Microbiol 78:2578–2585. doi:10.1128/AEM.07520-11 22307301 PMC3318799

[3] Croxen MA, Law RJ, Scholz R, Keeney KM, Wlodarska M, Finlay BB. 2013. Recent advances in understanding enteric pathogenic Escherichia coli. Clin Microbiol Rev 26:822–880. doi:10.1128/CMR.00022-13 24092857 PMC3811233

[4] Padola NL, Sanz ME, Blanco JE, Blanco M, Blanco J, Etcheverria AI, Arroyo GH, Usera MA, Parma AE. 2004. Serotypes and virulence genes of bovine Shigatoxigenic Escherichia coli (STEC) isolated from a feedlot in Argentina. Vet Microbiol 100:3–9. doi:10.1016/S0378-1135(03)00127-5 15135507

[5] Bai X, Mernelius S, Jernberg C, Einemo I-M, Monecke S, Ehricht R, Löfgren S, Matussek A. 2018. Shiga toxin-producing Escherichia coli infection in Jönköping County, Sweden: occurrence and molecular characteristics in correlation with clinical symptoms and duration of stx shedding. Front Cell Infect Microbiol 8:125. doi:10.3389/fcimb.2018.00125 29765909 PMC5939558

[6] Food and Agriculture Organization of the United Nations, World Health Organization. 2019. Shiga toxin-producing Escherichia coli (STEC) and food: attribution, characterization, and monitoring: meeting Report. Food Agric Org

[7] Sperandio V, Kaper JB, Bortolini MR, Neves BC, Keller R, Trabulsi LR. 1998. Characterization of the locus of enterocyte effacement (LEE) in different enteropathogenic Escherichia coli (EPEC) and Shiga-toxin producing Escherichia coli (STEC) serotypes. FEMS Microbiol Lett 164:133–139. doi:10.1111/j.1574-6968.1998.tb13078.x 9675859

[8] Colello R, Krüger A, Velez MV, Del Canto F, Etcheverría AI, Vidal R, Padola NL. 2019. Identification and detection of iha subtypes in LEE-negative Shiga toxin-producing Escherichia coli (STEC) strains isolated from humans, cattle and food. Heliyon 5:e03015. doi:10.1016/j.heliyon.2019.e03015 31879713 PMC6920203

[9] Kim J-S, Lee M-S, Kim JH. 2020. Recent updates on outbreaks of Shiga Toxin-producing Escherichia coli and its potential reservoirs. Front Cell Infect Microbiol 10:273. doi:10.3389/fcimb.2020.00273 32582571 PMC7287036

[10] Persad AK, LeJeune JT. 2014. Animal reservoirs of Shiga Toxin-producing Escherichia coli. Microbiol Spectr 2:EHEC–0027 doi:10.1128/microbiolspec.EHEC-0027-2014 26104194

[11] Kobuszewska A, Wysok B. 2024. Pathogenic bacteria in free-living birds, and its public health significance. Animals (Basel) 14:968. doi:10.3390/ani14060968 38540066 PMC10967383

[12] Ogden ID, MacRae M, Strachan NJC. 2005. Concentration and prevalence of Escherichia coli O157 in sheep faeces at pasture in Scotland. J Appl Microbiol 98:646–651. doi:10.1111/j.1365-2672.2004.02493.x 15715867

[13] Fremaux B, Prigent-Combaret C, Vernozy-Rozand C. 2008. Long-term survival of Shiga toxin-producing Escherichia coli in cattle effluents and environment: an updated review. Vet Microbiol 132:1–18. doi:10.1016/j.vetmic.2008.05.015 18586416

[14] González-Escalona N, Kase JA. 2019. Virulence gene profiles and phylogeny of Shiga toxin-positive Escherichia coli strains isolated from FDA regulated foods during 2010-2017. PLoS One 14:e0214620. doi:10.1371/journal.pone.0214620 30934002 PMC6443163

[15] Beer KD, Gargano JW, Roberts VA, Hill VR, Garrison LE, Kutty PK, Hilborn ED, Wade TJ, Fullerton KE, Yoder JS. 2015. Surveillance for waterborne disease outbreaks associated with drinking water — United States, 2011–2012. MMWR Morb Mortal Wkly Rep 64:842–848. doi:10.15585/mmwr.mm6431a2 26270059 PMC4584589

[16] Osińska A, Korzeniewska E, Korzeniowska-Kowal A, Wzorek A, Harnisz M, Jachimowicz P, Buta-Hubeny M, Zieliński W. 2022. The challenges in the identification of Escherichia coli from environmental samples and their genetic characterization. Environ Sci Pollut Res 30:11572–11583. doi:10.1007/s11356-022-22870-8 PMC9898413 36094711

[17] Umpiérrez A, Bado I, Oliver M, Acquistapace S, Etcheverría A, Padola NL, Vignoli R, Zunino P. 2017. Zoonotic potential and antibiotic resistance of Escherichia coli in Neonatal Calves in Uruguay. Microbes Environ 32:275–282. doi:10.1264/jsme2.ME17046 28904264 PMC5606698

[18] Umpiérrez Ana, Ernst D, Fernández M, Oliver M, Casaux ML, Caffarena RD, Schild C, Giannitti F, Fraga M, Zunino P. 2021. Virulence genes of Escherichia coli in diarrheic and healthy calves. Revista Argentina de Microbiología 53:34–38. doi:10.1016/j.ram.2020.04.004 32553726

[19] Ministerio de Salud Pública. 2012. Decreto N.° 41/012. Declárase de NotificacióN Obligatoria Un Listado de Enfermedades y Eventos Sanitarios. Available from: https://www.impo.com.uy/bases/decretos/41-2012

[20] Argentina.gob.ar. 2024. National Epidemiological Bulletin 2023. Available from: https://www.argentina.gob.ar/boletin-epidemiologico-nacional/boletines-2023

[21] Varela G, Chinen I, Gadea P, Miliwebsky E, Mota MI, González S, González G, Gugliada MJ, Carbonari CC, Algorta G, Bernadá M, Sabelli R, Pardo L, Rivas M, Schelotto F. 2008. Detection and characterization of Shiga toxin-producing Escherichia coli from clinical cases and food in Uruguay. Rev Argent Microbiol 40:93–100. 18705489

[22] Varela G, Batthyány L, Bianco MN, Pérez W, Pardo L, Algorta G, Robino L, Suárez R, Navarro A, Pírez MC, Schelotto F. 2015. Enteropathogens associated with acute diarrhea in children from households with high socioeconomic level in Uruguay. Int J Microbiol 2015:592953. doi:10.1155/2015/592953 25861274 PMC4377524

[23] Gadea MP, Deza N, Mota MI, Carbonari C, Robatto M, D’Astek B, Balseiro V, Bazet C, Rügnitz E, Livrelli V, Schelotto F, Rivas M, Varela G. 2012. Two cases of urinary tract infection caused by Shiga toxin-producing Escherichia coli O157:H7 strains. RevArgentMicrobiol 44 22997767

[24] Mota MI, Vázquez S, Cornejo C, D’Alessandro B, Braga V, Caetano A, Betancor L, Varela G. 2020. Does Shiga Toxin-producing Escherichia coli and Listeria monocytogenes contribute significantly to the burden of antimicrobial resistance in Uruguay? Front Vet Sci 7:583930. doi:10.3389/fvets.2020.583930 33240959 PMC7677299

[25] Umpiérrez A, Ernst D, Cardozo A, Torres A, Fernández M, Fragas M, Vignolo R, Bado I, Vidal R, Zunino P. 2022. Non-O157 Shiga toxin-producing Escherichia coli with potential harmful profiles to humans are isolated from the faeces of calves in Uruguay. Vet Sci 11:68. doi:10.3390/vetsci11020068

[26] Umpiérrez A, Ernst D, Fernández M, Oliver M, Casaux ML, Caffarena RD, Schild C, Giannitti F, Fraga M, Zunino P. 2021. Virulence genes of Escherichia coli in diarrheic and healthy calves. Rev Argent Microbiol 53:34–38. doi:10.1016/j.ram.2020.04.004 32553726

[27] Mussio P, Martínez I, Luzardo S, Navarro A, Leotta G, Varela G. 2023. Phenotypic and genotypic characterization of Shiga toxin-producing Escherichia coli strains recovered from bovine carcasses in Uruguay. Front Microbiol 14:1130170. doi:10.3389/fmicb.2023.1130170 36950166 PMC10025531

[28] Martínez de la Escalera G. 2025. Temporal and spatial changes of ecosystem health: case of los chanchos basin, Uruguay - The Rufford Foundation. Available from: https://www.rufford.org/projects/gabriela-mart%C3%ADnez-de-la-escalera/temporal-and-spatial-changes-ecosystem-health-case-los-chanchos-basin-uruguay/.Retrieved

[29] WEF. 1998. Standard methods for the examination of water and wastewater. 20th ed. American Public Health Association, Washington, DC.

[30] ISO. 2012. ISO/TS 13136:2012. Microbiology of food and animal feed—Real-time polymerase chain reaction (PCR)-based method for the detection of food-borne pathogens—Horizontal method for the detection of Shiga toxin-producing Escherichia coli (STEC) and the determination of O157, O111, O26, O103 and O145 serogroups. International Organization for Standardization, Geneva, Switzerland. Geneva, Switzerland International Organization for Standardization

[31] González Revello A. 2020. Generación de métodos basados en genómica para detectar microrganismos nocivos en sistemas acuáticos.Tesis

[32] Octavia S, Lan R. 2014. The Family Enterobacteriaceae, p 225–286. In Rosenberg E, DeLong EF, Lory S, Stackebrandt E, Thompson F (ed), The Prokaryotes. Springer, Berlin, Heidelberg.

[33] Clinical and Laboratory Standards Institute (CLSI). 2025. CLSI M100 Performance Standards for Antimicrobial Susceptibility Testing. Clinical and Laboratory Standards Institute, Wayne, PA.

[34] Microbes NG. 2025 Whole genome sequencing. Available from: https://microbesng.com/whole-genome-sequencing/

[35] 2020. Galaxy Project. Available from: https://aries.iss.it

[36] Knijn A, Michelacci V, Gigliucci F, Tozzoli R, Chiani P, Minelli F, Scavia G, Ventola E, Morabito S. 2023. IRIDA-ARIES genomics, a key player in the one health surveillance of diseases caused by infectious agents in Italy. Front Public Health 11:1151568. doi:10.3389/fpubh.2023.1151568 37361153 PMC10289303

[37] Bittencourt SA. 2010. FastQC: a quality control tool for high throughput sequence data*.* Available from: https://www.scienceopen.com/document?vid=de674375-ab83-4595-afa9-4c8aa9e4e736

[38] Thangadurai D, Sangeetha J. 2015. Genomics and proteomics: principles, technologies, and applications. CRC Press.

[39] Hasman H, Saputra D, Sicheritz-Ponten T, Lund O, Svendsen CA, Frimodt-Møller N, Aarestrup FM. 2014. Rapid whole-genome sequencing for detection and characterization of microorganisms directly from clinical samples. J Clin Microbiol 52:3136–3136. doi:10.1128/JCM.01369-14 PMC3911411 24172157

[40] Bankevich A, Nurk S, Antipov D, Gurevich AA, Dvorkin M, Kulikov AS, Lesin VM, Nikolenko SI, Pham S, Prjibelski AD, Pyshkin AV, Sirotkin AV, Vyahhi N, Tesler G, Alekseyev MA, Pevzner PA. 2012. SPAdes: a new genome assembly algorithm and its applications to single-cell sequencing. J Comput Biol 19:455–477. doi:10.1089/cmb.2012.0021 22506599 PMC3342519

[41] Gurevich A, Saveliev V, Vyahhi N, Tesler G. 2013. QUAST: quality assessment tool for genome assemblies. Bioinformatics 29:1072–1075. doi:10.1093/bioinformatics/btt086 23422339 PMC3624806

[42] Joensen KG, Tetzschner AMM, Iguchi A, Aarestrup FM, Scheutz F. 2015. Rapid and easy in silico serotyping of Escherichia coli isolates by use of whole-genome sequencing data. J Clin Microbiol 53:2410–2426. doi:10.1128/JCM.00008-15 25972421 PMC4508402

[43] Seemann T. 2016. ABRicate: mass screening of contigs for antimicrobial and virulence genes. Available from: https://github.com/tseemann/abricate

[44] Seemann T. 2014. Prokka: rapid prokaryotic genome annotation. Bioinformatics 30:2068–2069. doi:10.1093/bioinformatics/btu153 24642063

[45] Franzin FM, Sircili MP. 2015. Locus of enterocyte effacement: a pathogenicity island involved in the virulence of enteropathogenic and enterohemorragic Escherichia coli subjected to a complex network of gene regulation. Biomed Res Int 2015:534738. doi:10.1155/2015/534738 25710006 PMC4332760

[46] Clermont O, Dixit OVA, Vangchhia B, Condamine B, Dion S, Bridier-Nahmias A, Denamur E, Gordon D. 2019. Characterization and rapid identification of phylogroup G in Escherichia coli, a lineage with high virulence and antibiotic resistance potential. Environ Microbiol 21:3107–3117. doi:10.1111/1462-2920.14713 31188527

[47] Kearse M, Moir R, Wilson A, Stones-Havas S, Cheung M, Sturrock S, Buxton S, Cooper A, Markowitz S, Duran C, Thierer T, Ashton B, Meintjes P, Drummond A. 2012. Geneious Basic: an integrated and extendable desktop software platform for the organization and analysis of sequence data. Bioinformatics 28:1647–1649. doi:10.1093/bioinformatics/bts199 22543367 PMC3371832

[48] Montero DA, Velasco J, Del Canto F, Puente JL, Padola NL, Rasko DA, Farfán M, Salazar JC, Vidal R. 2017. Locus of adhesion and autoaggregation (LAA), a pathogenicity island present in emerging Shiga Toxin-producing Escherichia coli strains. Sci Rep 7:7011. doi:10.1038/s41598-017-06999-y 28765569 PMC5539235

[49] Nüesch-Inderbinen M, Stevens MJA, Cernela N, Müller A, Biggel M, Stephan R. 2021. Distribution of virulence factors, antimicrobial resistance genes and phylogenetic relatedness among Shiga toxin-producing Escherichia coli serogroup O91 from human infections. Int J Med Microbiol 311:151541. doi:10.1016/j.ijmm.2021.151541 34757276

[50] Darling AE, Mau B, Perna NT. 2009. Progressive mauve: multiple alignment of genomes with gene flux and rearrangement. BMC Bioinformatics

[51] Sullivan MJ, Petty NK, Beatson SA. 2011. Easyfig: a genome comparison visualizer. Bioinformatics 27:1009–1010. doi:10.1093/bioinformatics/btr039 21278367 PMC3065679

[52] Page AJ, Cummins CA, Hunt M, Wong VK, Reuter S, Holden MTG, Fookes M, Falush D, Keane JA, Parkhill J. 2015. Roary: rapid large-scale prokaryote pan genome analysis. Bioinformatics 31:3691–3693. doi:10.1093/bioinformatics/btv421 26198102 PMC4817141

[53] Page AJ, Taylor B, Delaney AJ, Soares J, Seemann T, Keane JA, Harris SR. 2016. SNP-sites: rapid efficient extraction of SNPs from multi-FASTA alignments. MicrobGenom 2:e000056. doi:10.1099/mgen.0.000056 PMC5320690 28348851

[54] Nguyen L-T, Schmidt HA, von Haeseler A, Minh BQ. 2015. IQ-TREE: a fast and effective stochastic algorithm for estimating maximum-likelihood phylogenies. Mol Biol Evol 32:268–274. doi:10.1093/molbev/msu300 25371430 PMC4271533

[55] 2018. FigTree*.* Available from: http://tree.bio.ed.ac.uk/software/figtree

[56] 2024. Letunic I. iTOL: interactive tree of life. Available from: https://itol.embl.de/

[57] Silva M, Machado MP, Silva DN, Rossi M, Moran-Gilad J, Santos S, Ramirez M, Carriço JA. 2018. chewBBACA: A complete suite for gene-by-gene schema creation and strain identification. Microb Genom 4:e000166. doi:10.1099/mgen.0.000166 29543149 PMC5885018

[58] Saini P, Bandsode V, Singh A, Mendem SK, Semmler T, Alam M, Ahmed N. 2024. Genomic insights into virulence, antimicrobial resistance, and adaptation acumen of Escherichia coli isolated from an urban environment. mBio 15:e0354523. doi:10.1128/mbio.03545-23 38376265 PMC10936179

[59] Melton-Celsa AR. 2014. Shiga Toxin (Stx) classification, structure, and function. Microbiol Spectr 2:EHEC–0024 doi:10.1128/microbiolspec.EHEC-0024-2013 PMC4270005 25530917

[60] Bai X, Zhang J, Hua Y, Jernberg C, Xiong Y, French N, Löfgren S, Hedenström I, Ambikan A, Mernelius S, Matussek A. 2021. Genomic insights into clinical Shiga Toxin-producing Escherichia coli strains: a 15-year period survey in Jönköping, Sweden. Front Microbiol 12:627861. doi:10.3389/fmicb.2021.627861 33613494 PMC7893091

[61] Capps KM, Ludwig JB, Shridhar PB, Shi X, Roberts E, DebRoy C, Cernicchiaro N, Phebus RK, Bai J, Nagaraja TG. 2021. Identification, Shiga toxin subtypes and prevalence of minor serogroups of Shiga toxin-producing Escherichia coli in feedlot cattle feces. Sci Rep 11:8601. doi:10.1038/s41598-021-87544-w 33883564 PMC8060326

[62] Dias D, Costa S, Fonseca C, Baraúna R, Caetano T, Mendo S. 2022. Pathogenicity of Shiga toxin-producing Escherichia coli (STEC) from wildlife: should we care? Sci Total Environ 812:152324. doi:10.1016/j.scitotenv.2021.152324 34915011

[63] Rounds JM, Rigdon CE, Muhl LJ, Forstner M, Danzeisen GT, Koziol BS, Taylor C, Shaw BT, Short GL, Smith KE. 2012. Non-O157 Shiga toxin-producing Escherichia coli associated with venison. Emerg Infect Dis 18:279–282. doi:10.3201/eid1802.110855 22305114 PMC3310449

[64] Bertelloni F, Cilia G, Bogi S, Ebani VV, Turini L, Nuvoloni R, Cerri D, Fratini F, Turchi B. 2020. Pathotypes and antimicrobial susceptibility of Escherichia Coli isolated from wild boar (Sus scrofa) in Tuscany. Animals (Basel) 10:744. doi:10.3390/ani10040744 32344604 PMC7222796

[65] Ministerio de Ambiente. 2024. Decreto N° 198/024: Excepciones de la prohibición de caza nocturna. Montevideo, Uruguay. Disponible En. Available from: https://www.gub.uy/ministerio-ambiente/institucional/normativa/decreto-n-198024-excepciones-prohibicion-caza-nocturna

[66] Gobierno de Uruguay. 1994. Decreto 315/94, Artículo 13. Available from: https://www.impo.com.uy/

[67] Malesa R, Pierneef R, Magwedere K, Mafuna T, Matle I. 2024. Genomic characterisation of generic Escherichia coli from food-producing animals and products of animal origin in South Africa. Front Bacteriol 3:1432292. doi:10.3389/fbrio.2024.1432292

[68] Murase K, Ooka T, Iguchi A, Ogura Y, Nakayama K, Asadulghani M, Islam MR, Hiyoshi H, Kodama T, Beutin L, Hayashi T. 2012. Haemolysin E- and enterohaemolysin-derived haemolytic activity of O55/O157 strains and other Escherichia coli lineages. Microbiology (Reading) 158:746–758. doi:10.1099/mic.0.054775-0 22194351

[69] Fu S, Bai X, Fan R, Sun H, Xu Y, Xiong Y. 2018. Genetic diversity of the enterohaemolysin gene (ehxA) in non-O157 Shiga toxin-producing Escherichia coli strains in China. Sci Rep 8:1–8. doi:10.1038/s41598-018-22699-7 29523817 PMC5844952

[70] Huerta-Cantillo J, Chavez-Dueñas L, Zaidi MB, Estrada-García T, Navarro-Garcia F. 2025. Cytolethal distending toxin-producing Escherichia coli clinical isolates from Mexican children harbor different CDT types causing CDT-induced epithelial pathological phenotypes. Med Microbiol Immunol 214:7. doi:10.1007/s00430-025-00816-4 39894890 PMC11788229

[71] Paton AW, Paton JC. 2001. Pathogenesis and diagnosis of Shiga toxin-producing Escherichia coli infections. Clin MicrobiolRev 14:597–624. doi:10.1128/CMR.14.3.597-624.2001 PMC88891 9665978

[72] Michelacci V, Montalbano Di Filippo M, Gigliucci F, Arancia S, Chiani P, Minelli F, Roosens NHC, De Keersmaecker SCJ, Bogaerts B, Vanneste K, Morabito S. 2022. Population analysis of O26 Shiga toxin-producing Escherichia coli causing hemolytic uremic syndrome in Italy, 1989–2020, through whole genome sequencing. Front Cell Infect Microbiol 12:842508. doi:10.3389/fcimb.2022.842508 35223557 PMC8864317

[73] Hancock V, Witsø IL, Klemm P. 2011. Biofilm formation as a function of adhesin, growth medium, substratum and strain type. Int J Med Microbiol 301:570–576. doi:10.1016/j.ijmm.2011.04.018 21646046

[74] Schiebel J, Böhm A, Nitschke J, Burdukiewicz M, Weinreich J, Ali A, Roggenbuck D, Rödiger S, Schierack P. 2017. Genotypic and phenotypic characteristics associated with biofilm formation by human clinical Escherichia coli isolates of different pathotypes. Appl Environ Microbiol 83:e00100–17. doi:10.1128/AEM.01660-17 PMC5717203 28986371

[75] Wang L, Bai X, Ylinen E, Zhang J, Saxén H, Matussek A. 2023. Genotypic and phenotypic characteristics associated with biofilm formation by human clinical Escherichia coli isolates of different pathotypes. Toxins (Basel) 15:669. doi:10.3390/toxins15120669 38133173 PMC10748226

[76] Moeinirad M, Douraghi M, Rahimi Foroushani A, Sanikhani R, Soltan Dallal MM. 2021. Molecular characterization and prevalence of virulence factor genes of Shiga toxin-producing Escherichia coli (STEC) isolated from diarrheic children. Gene Rep 25:101379. doi:10.1016/j.genrep.2021.101379

[77] Segura A, Auffret P, Bibbal D, Bertoni M, Durand A, Jubelin G, Kérourédan M, Brugère H, Bertin Y, Forano E. 2018. Factors involved in the persistence of a Shiga Toxin-producing Escherichia coli O157:H7 strain in Bovine feces and gastro-intestinal content. Front Microbiol 9:375. doi:10.3389/fmicb.2018.00375 29593666 PMC5854682

[78] Awosile B, Fritzler J, Levent G, Rahman MK, Ajulo S, Daniel I, Tasnim Y, Sarkar S. 2023. Genomic characterization of fecal Escherichia coli isolates with reduced susceptibility to beta-lactam antimicrobials from wild hogs and coyotes. Pathogens 12:929. doi:10.3390/pathogens12070929 37513776 PMC10383658

[79] Sano K, Kobayashi H, Chuta H, Matsuyoshi N, Kato Y, Ogasawara H. 2023. CsgI (YccT) is a novel inhibitor of curli fimbriae formation in Escherichia coli preventing CsgA polymerization and curli gene expression. IJMS 24:4357. doi:10.3390/ijms24054357 36901788 PMC10002515

[80] Teng C-H, Tseng Y-T, Maruvada R, Pearce D, Xie Y, Paul-Satyaseela M, Kim KS. 2010. NlpI contributes to Escherichia coli K1 strain RS218 interaction with human brain microvascular endothelial cells. Infect Immun 78:3090–3096. doi:10.1128/IAI.00034-10 20421385 PMC2897387

[81] Tseng Y, Wang S-W, Kim KS, Wang Y-H, Yao Y, Chen C-C, Chiang C-W, Hsieh P-C, Teng C-H. 2012. NlpI facilitates deposition of C4bp on Escherichia coli by blocking classical complement-mediated killing, which results in high-level bacteremia. Infect Immun 80:3669–3678. doi:10.1128/IAI.00320-12 22802341 PMC3457581

[82] Geurtsen J, de Been M, Weerdenburg E, Zomer A, McNally A, Poolman J. 2022. Genomics and pathotypes of the many faces of Escherichia coli. FEMS Microbiol Rev 46:fuac019. doi:10.1093/femsre/fuac031 35749579 PMC9629502

[83] Byarugaba DK, Wokorach G, Alafi S, Erima B, Najjuka F, Mworozi EA, Kibuuka H, Wabwire-Mangen F. 2023. Whole genome sequencing reveals high genetic diversity, diverse repertoire of virulence-associated genes and limited antibiotic resistance genes among commensal Escherichia coli from food animals in Uganda. Microorganisms 11:1868. doi:10.3390/microorganisms11081868 37630428 PMC10457813

[84] Tramonti A, De Canio M, Delany I, Scarlato V, De Biase D. 2006. Mechanisms of transcription activation exerted by GadX and GadW at the gadA and gadBC gene promoters of the glutamate-based acid resistance system in Escherichia coli. J Bacteriol 188:8118–8127. doi:10.1128/JB.01044-06 16980449 PMC1698215

[85] Al-Sarawi HA, Habibi N, Uddin S, Jha AN, Al-Sarawi MA, Lyons BP. 2023. Antibiotic resistance mediated by Escherichia coli in Kuwait marine environment as revealed through genomic analysis. Antibiotics (Basel) 12:1366. doi:10.3390/antibiotics12091366 37760663 PMC10525739

[86] Thaker M, Spanogiannopoulos P, Wright GD. 2010. The tetracycline resistome. Cell Mol Life Sci 67:419–431. doi:10.1007/s00018-009-0172-6 19862477 PMC11115633

[87] Nonaka L, Suzuki S. 2002. New Mg2+-dependent oxytetracycline resistance determinant tet(34) in Vibrio isolates from marine fish intestinal contents. Antimicrob Agents Chemother 46:1550–1552. doi:10.1128/AAC.46.5.1550-1552.2002 11959596 PMC127156

[88] Zhang T, Zhang M, Zhang X, Fang HH. 2009. Tetracycline resistance genes and tetracycline resistant lactose-fermenting Enterobacteriaceae in activated sludge of sewage treatment plants. Environ Sci Technol 43:3455–3460. doi:10.1021/es803309m 19544839

[89] Blake KS, Xue Y-P, Gillespie VJ, Fishbein SRS, Tolia NH, Wencewicz TA, Dantas G. 2025. The tetracycline resistome is shaped by selection for specific resistance mechanisms by each antibiotic generation. Nat Commun 16:1–14. doi:10.1038/s41467-025-56425-5 39920134 PMC11806011

[90] ResFinder. 2020 Center for genomic epidemiology. Available from: http://genepi.food.dtu.dk/resfinder

[91] Tran ML, Delannoy S, Fach P. 2025. Enhancing detection of STEC in the meat industry: insights into virulence of priority STEC. Front Microbiol 16:1543686. doi:10.3389/fmicb.2025.1543686 40012779 PMC11860885

[92] Pintara A, Jennison A, Rathnayake IU, Mellor G, Huygens F. 2020. Core and accessory genome comparison of Australian and international strains of o157 shiga toxin-producing Escherichia coli Front Microbiol 11:566415. doi:10.3389/fmicb.2020.566415 33013798 PMC7498637

[93] Lauzi S, Luzzago C, Chiani P, Michelacci V, Knijn A, Pedrotti L, Corlatti L, Buccheri Pederzoli C, Scavia G, Morabito S, Tozzoli R. 2022. Free-ranging red deer (Cervus elaphus) as carriers of potentially zoonotic Shiga toxin-producing Escherichia coli. Transbound Emerg Dis 69:1902–1911. doi:10.1111/tbed.14178 34080316 PMC9540879

[94] Marzano MA, Riera CM, Garavano J, Ruiz-Holgado VA. 2021. Calidad microbiológica de la carne picada y detección de patógenos en muestras ambientales de carnicerías de la ciudad de Tandil, provincia de Buenos Aires. Argentina. Rev Argent Microbiol 1:311–317. doi:10.1016/j.ram.2021.05.001 34556377

