# Supplementary material for: STEC in the natural environment of Uruguay: genomic surveillance and environmental circulation in the framework of One Health
Source: Microbiol Spectr. 2025 Nov 28;14(1):e03153-25. doi: 10.1128/spectrum.03153-25 (PMC12772381; doi:10.1128/spectrum.03153-25)
Supplement: Figure S1 and Table S1 — Figure S1: Geographical localization of Villa Serrana village and stream "Los Chanchos" in Uruguay, coverage and land uses, sample sites. Table S1: LEE-negative STEC used to generate the Core genome SNPs tree. [file spectrum.03153-25-s0001.pdf]

Figure S1

Geographical localization of Villa Serrana village and stream "Los Chanchos" in Uruguay, coverage and land uses, sample sites.

Supplementary Table

LEE-negative STEC used to generate the Core genome SNPs tree.

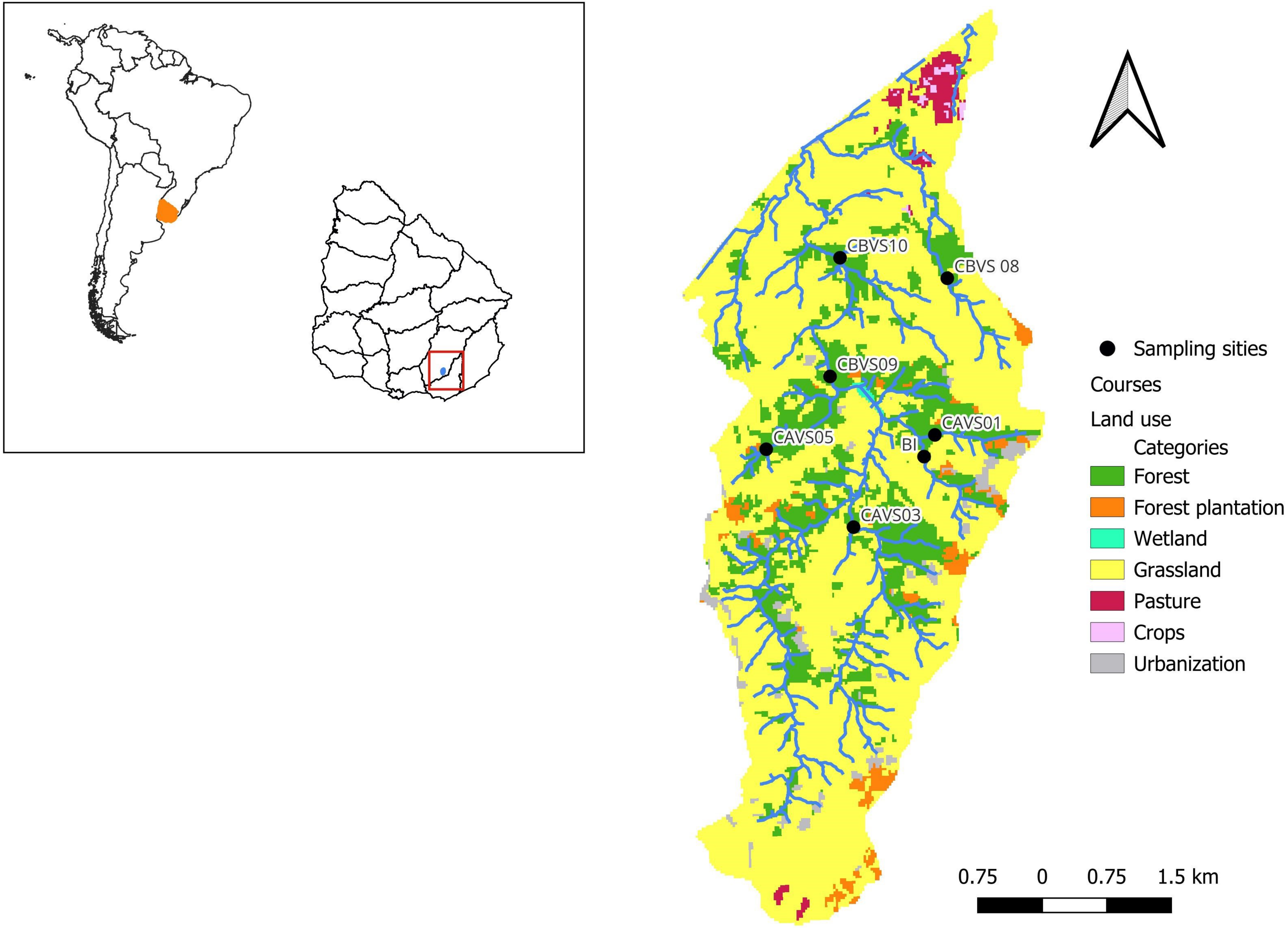

CBVS10
CBVS 08
CBVS09
CAVS01
BI
CAVS05
CAVS03
Sampling sities
Courses
Land use
Categories
Forest
Forest plantation
Wetland
Grassland
Pasture
Crops
Urbanization
0.75 0 0.75 1.5 km

ID strain	Serogroup / Serotype	MultiLocus Sequence Typing	Origin / Host	Origin / Sample
211_1	O103:H42	ST-692	Bovine	Feces
HW 1-3	O22:H8	ST-446	Food sample	Hamburguer
348_3	O46	ST-154	Bovine	Feces
AM 162-1	O39:H49	ST-2520	Bovine	Feces
175_1	O156:H-	ST-441	Bovine	Feces
115_4	O171:H2	ST-332	Bovine	Feces
116_1	O20:H19	ST-443	Bovine	Feces
HT 1-6	O20:H19	ST-443	Food sample	Hamburguer
208_3	O139:H19	ST-1125	Bovine	Feces
180-3-4r	O178:H19	ST-192	Food sample	Chicken burguer
IMP-886	O20:H19	ST-443	Food sample	Beef trim
218_8	O163:H9	ST-679	Bovine	Feces
IMP-871	O163:H19	ST-679	Food sample	Beef trim
CM 15-2	O8:H16	ST-2217	Food sample	Ground beef
30M	O8:H19	ST-1431	Food sample	Ground beef
45-2-4	O8:H19	ST-1431	Bovine	Feces
AP 32-1	O117:H7	ST-2387	NA	-
6_6	O130:H11	ST-297	Bovine	Feces
AP 31-1	O141:H8	ST-297	NA	-
E044-00	O113:H21	ST-56	Human	Feces
E045-00	O113:H21	ST-56	Human	Feces
E042-00	O113:H21	ST-56	Human	Feces
E043-00	O113:H21	ST-56	Human	Feces
E046-00	O113:H21	ST-56	Human	Feces
365_1	O7	ST-58	Bovine	Feces
V07-4-4	O91:H21	ST-442	Bovine	Feces
AP 16-1	O91:H21	ST-442	NA	-
47-1-1	O91:H21	ST-442	Bovine	Feces
FO 130	O91:H21	ST-442	NA	-
1/5/2001	O91:H21	ST-442	Bovine	Feces
58_3	O174:H21	ST-677	Bovine	Feces
26_1	O2	ST-2715	Bovine	Feces
MOD1-EC5931	-	ST-993	Sus scrofa	Large intestine
MOD1-EC5170	-	ST-6245	Sus scrofa	Feces
11229	-	ST-446	us nippon yeso	Feces
MOD1-EC5167	-	ST-388	Sus scrofa	Feces
MOD1-EC6230	O112:H21	ST-6229	ocoileus virginia	Small intestine
F339	O20:H19	ST-5285	Sus scrofa	Feces
F313	O163:H-	ST-5285	Sus scrofa	Feces
C316	O163:H32	ST-5285	Sus scrofa	Feces
C104	O174:H2	ST-1148	Sus scrofa	Feces
MOD1-EC5271	-	ST-641	Sus domesticus	Feces
MOD1-EC6953	-	ST-1423	otragus oreotra	Feces
15484-1	O84	ST-2385	Enviroment	Water-pond
MOD1-EC5169	-	ST-297	Sus scrofa	Feces
MOD1-EC6202	O187:H52	ST-642	ocoileus virginia	Feces
RM7807	O113:H21	ST-5975	Sus scrofa	Feces
RM7806	O113:H21	ST-5975	Sus scrofa	Feces
RM7788	O113:H21	ST-5975	Enviroment	Water

13950-1	O38	ST-56	Enviroment	Water-pond
MOD1-EC6845	-	ST-13	Gazella cuvieri	Rectum
MOD1-EC5267	-	ST-33	Sus domesticus	Feces
P.231	O91:H21	ST-442	Pig	Feces
MOD1-EC6997	-	ST-442	Pig	Feces
16309	-	ST-5597	us nippon yeso	Feces
MOD1-EC1636	O138:H14	ST-42	Sus scrofa	Feces
MOD1-EC5751	-	ST-42	Sus scrofa	Ileum
MOD1-EC6672	O139:H6	ST-955	Pig	Intestine
4709-1	O136	ST-329	Deer	Feces
S1191	O139:H1	ST-1260	Pig	Feces
MOD1-EC1638	O101:H9	ST-10	Sus scrofa	feces
MOD1-EC1637	O101:H9	ST-10	Sus scrofa	Feces
15166-1	O109	Unknown	Enviroment	Water-pond
MOD1-EC1681	O153:H31	ST-101	Bos taurus	Feces
1553-1	O121	Unknown	Bovine	Feces
2009C-3299	O121:H7	ST-5082	NA	-
F313	O163:H-	ST-5285	Sus scrofa	Feces
C316	O163:H32	ST-5285	Sus scrofa	Feces
CFSAN026773	O6	Unknown	Bovine	NA
MOD1-EC2446	O6:H34	ST-5559	Bos taurus	Feces
MOD1-EC5271	-	ST-641	Sus domesticus	Feces
MOD1-EC6563	-	ST-5082	Equine	Heart
MOD1-EC6865	OX21:H51	ST-679	Bovine	Feces
9388-1	O163	ST-679	Bovine	Feces
JAN-020	O163:H19	ST-679	Food sample	Beef trim
MOD1-EC6565	O22:H1	ST-223	Bovine	Feces
MOD1-EC6572	O4:H7	ST-223	Bovine	Feces
MOD1-EC6858	-	ST-677	Bovine	Feces
MOD1-EC6027	O174:H36	ST-677	Food sample	-
MOD1-EC1920	O174:H21	ST-677	Food sample	-
MOD1-EC1632	O174:H21	ST-677	Human	Feces
MOD1-EC1633	O174:H21	ST-677	Human	Feces
MOD1-EC1944	O174:H21	ST-677	Bos Taurus	Feces
220/00	O174:H21	ST-677	Human	fecees
STEC 200	O174:H21	ST-677	Human	Feces
MOD1-EC1650	O174:H21	ST-677	Human	Feces
031.	O174:H21	ST-677	Human	Bowel
OLC-2141	O174:H21	ST-677	NA	NA
OLC-1265	O174:H21	ST-677	NA	NA
MOD1-EC1667	O174:H21	ST-677	NA	-
03-3269.	O174:H21	ST-677	NA	-
STEC 3098	O174:H21	ST-677	Human	Feces
MOD1-EC1985	O174:H21	ST-677	Human	Feces
MOD1-EC1657	O174:H21	ST-677	Human	Feces
03-3458.	O119:H4	ST-117	NA	-

Country	Year	Shiga-toxin subtype(s)	Accesion Number
Chile	NA	stx1a, stx2d	QESC00000000
Argentina	1998	stx1a, stx2a	QESK00000000
Chile	NA	stx1a, stx2a	QESI00000000
Argentina	1998	stx2a	QESJ00000000
Chile	NA	stx2a	QERN00000000
Chile	NA	stx2c	QERL00000000
Chile	NA	stx1a, stx2a	QESM00000000
Argentina	1998	stx1a, stx2a	QESL00000000
Chile	NA	stx2c	QERP00000000
Argentina	2007	stx2c	QERJ00000000
Uruguay	2005	stx1a, stx2a	NWAF00000000
Chile	NA	stx2a	QERM00000000
Uruguay	2004	stx2a	NWAG00000000
Argentina	1998	stx1a	QESP00000000
Argentina	1998	stx2a	QESO00000000
Argentina	2009	stx2a	QESN00000000
Argentina	1998	stx2c	QERR00000000
Chile	NA	stx2d	QERQ00000000
Argentina	1998	stx1, stx2a	QERO00000000
Chile	2000	stx2c	QERZ00000000
Chile	2000	stx2c	QERY00000000
Chile	2000	stx2c	QERU00000000
Chile	2000	stx2c	QERT00000000
Chile	2000	stx2c	QERS00000000
Chile	NA	stx2a	QESQ00000000
Argentina	2008	stx2d	QESH00000000
Argentina	1998	stx2d	QESG00000000
Argentina	2009	stx2a	QESF00000000
Argentina	2001	stx2a	QESE00000000
Argentina	2010	stx2a	QESD00000000
Chile	NA	stx2c	QERK00000000
Chile	NA	stx2g	QESR00000000
Puerto Rico	1985	stx2e	NKUO00000000
USA	2008	stx2a	NOFM00000000
Japan	2011	stx2b	BDLJ00000000
USA	2008	stx2d	NOFN00000000
USA	1986	stx1c, stx2b	NNYC00000000
USA	2000	stx1a	NWAN00000000
USA	2001	stx1a	NWAQ00000000
USA	2014	stx1a	NWAV00000000
USA	2014	stx2e	NWAZ00000000
USA	2000	stx2e	NLPY00000000
USA	1998	stx2b	NONS00000000
USA	1999	stx1a, stx2a	LHCN00000000
USA	2008	stx1a	NTPO00000000
USA	2013	stx1c	NTOK00000000
USA	2008	stx2a, stx2a	NWVQ00000000
USA	2008	stx2a, stx2a	NWVR00000000
USA	2008	stx2a, stx2a	NWVS00000000

USA	1999	stx2a	LDDU00000000
USA	1996	stx1c, stx2b	NOQN00000000
China	2000	stx1a	NLQC00000000
USA	NA	stx2d	JNNL00000000
USA	1975	stx1c	NOMF00000000
Japan	2016	stx1a	BDLM00000000
United Kingdom	NA	stx2e	NJZI00000000
USA	1982	stx2e	NODR00000000
USA	1991	stx2e	NNTG00000000
USA	1999	stx1a	LGZN00000000
USA	NA	stx2e	AFEA00000000
Italy	NA	stx2e	NJUY00000000
USA	NA	stx2e	NJUZ00000000
USA	1999	stx1a	LDCY00000000
Canada	1992	stx1a	NJUI00000000
USA	1999	stx1d	LDCX00000000
USA	NA	stx1d	JHHC00000000
USA	2001	stx1a	NWAQ00000000
USA	2014	stx1a	NWAV00000000
USA	1999	stx1a, stx2a	LDDW00000000
USA	2012	stx2c	NJSO00000000
USA	2000	stx2e	NLPY00000000
India	1990	stx1d	NMMS00000000
USA	1996	stx2a	NOPW00000000
USA	1999	stx1a, stx2a	LDDH00000000
USA	2006	stx2d	NWAE00000000
Canada	1990	stx2a	NMOH00000000
Canada	1999	stx2a	NMOE00000000
USA	1996	stx2c	NOQC00000000
USA	2010	stx2c	NMEN00000000
USA	1989	stx2c	NKNV00000000
Canada	1988	stx2c	NJVD00000000
Canada	1988	stx2c	NJVC00000000
Canada	1988	stx2c	NJTK00000000
Germany	2000	stx2c	APWX00000000
Netherlands	2013	stx2c	LNZL00000000
USA	1993	stx2b, stx2c	NJUU00000000
USA	1992	stx2c, stx2b	AFEX00000000
Canada	2015	stx2b, stx2d	NENN00000000
Canada	2014	stx2b, stx2d	NEOH00000000
Germany	2001	stx2c	NKOS00000000
USA	NA	stx2d	JHNW00000000
Netherlands	2014	stx2c	LPUM00000000
USA	2001	stx2c	NJXY00000000
USA	1998	stx2c	NJZD00000000
USA	NA	stx1a, stx2a	JHNV00000000
